# Using linked Hospital Episode Statistics data to aid the handling of non-response and restore sample representativeness in the 1958 National Child Development Study.

**DOI:** 10.23889/ijpds.v7i3.1997

**Published:** 2022-08-25

**Authors:** Nasir Rajah, Lisa Calderwood, Bianca De Stavola, Katie Harron, George Ploubidis, Richard Silverwood

**Affiliations:** 1 Centre for Longitudinal Studies, UCL Social Research Institute, University College London; 2 Population, Policy & Practice Department, UCL Great Ormond Street Institute of Child Health, University College London

## Objectives

There is growing interest in whether linked administrative data have the potential to aid analyses subject to missing data in cohort studies. We aimed to identify predictors of cohort non-response in linked administrative data and examine whether inclusion of these variables in principled methods for missing data handling can help restore sample representativeness.

## Approach

Using linked 1958 National Child Development Study (NCDS) and Hospital Episode Statistics (HES) data, we applied a multi-stage data-driven approach to identify HES variable which are predictive of non-response at the age 55 sweep of NCDS. We then included these variables as auxiliary variables in multiple imputation (MI) analyses to see if they helped restore sample representativeness in terms of early life variables which were essentially fully observed in NCDS (mother’s husband’s social class at birth, cognitive ability at age 7) and relative to external population data (educational qualifications at age 55, marital status at age 55).

## Results

We took as our starting point 57 variables derived from HES data based on the presence or number of different types of appointments/admissions, diagnostic codes and treatment codes. After application of our multi-stage data-driven approach we identified five HES variables that were predictive of non-response at age 55 in NCDS. For example, cohort members who had been treated for adult mental illness were almost 3 times as likely to be non-respondents (risk ratio 2.81; 95% confidence interval 2.05, 3.86). Inclusion of these variables in MI analyses did help restore sample representativeness. However, there was no additional gain in sample representativeness relative to analyses using only previously identified survey predictors of non-response (i.e. NCDS rather than HES variables).

## Conclusion

In our applications, inclusion of HES predictors of NCDS non-response in analyses did not improve sample representativeness beyond that possible using survey variables alone. Whilst this finding may not extend to other analyses or NCDS sweeps, it highlights the utility of survey variables in handling non-response.

